# Influence of Match Congestion on Performances in the National Basketball Association

**DOI:** 10.3389/fpsyg.2021.630769

**Published:** 2021-02-17

**Authors:** Jianzhe Yang, Chao Wu, Changjing Zhou, Shaoliang Zhang, Anthony S. Leicht, Miguel-Ángel Gomez

**Affiliations:** ^1^Department of Physical Education, Hohai University, Changzhou, China; ^2^Department of Physical Education, University of International Business and Economics, Beijing, China; ^3^School of Physical Education and Sport Training, Shanghai University of Sport, Shanghai, China; ^4^Division of Sport Science and Physical Education, Tsinghua University, Beijing, China; ^5^Sport and Exercise Science, James Cook University, Townsville, QLD, Australia; ^6^Facultad de Ciencias de la Actividad Física y del Deporte (INEF), Universidad Politécnica de Madrid, Madrid, Spain

**Keywords:** match schedule, performance analysis, elite sport, regression analysis, national basketball association

## Abstract

The ability to recover from official match-play across a single and multiple matches is often considered a key factor in subsequent performance for modern professional basketball. The aims of this study were to: (i) explore the differences in match performances between different match congestion cycles (i.e., matches separated by zero, one, or two or greater days of rest); and (ii) identify the key performance indicators (KPIs) discriminating between winning and losing during different match congestion cycles. The current study indicated that scoring close to (i.e., within the paint) (ES = 0.08) or very far away (i.e., Three-point, ES = 0.05) was significantly greater for winning matches separated by 1- and 2-days of rest compared to consecutive matches (i.e., 0 rest days between matches). Additionally, shooting efficiency (*P* < 0.001), and attaining Defensive Rebounds (*P* < 0.001) and Steals (*P* < 0.001), were significant offensive and defensive KPIs that differentiated winning and losing teams. Similarly, opponent quality and match pace were important situational variables that affected match outcome during different match congestion cycles. While match location had an impact on winning following 1- and 2-days of rest, it had no impact for back-to-back matches (i.e., 0 days between matches). The current results will support coaches' offensive, defensive and recovery strategies during various match congestion cycles for a greater probability of winning NBA matches.

## Introduction

The National Basketball Association (NBA) is the pre-eminent men's basketball league in the world and one of the four profitable and professional sports leagues in North America. Originating in 1946, the NBA currently has 30 teams with two conferences of three divisions that undertake significant travel for matches (Nutting, 2010). The playing schedule for all NBA teams consists of travel across four time zones that poses substantial challenges for athletes (Sampaio et al., 2015b). In contrast, teams from the National Football League travel similar distances and across zones but only compete once a week (Nutting, 2010). Major League Baseball teams compete in more matches but spend 4–5 days in each city and therefore have less congested travel schedules (Nutting, 2010). The NBA teams compete, on average, in three matches a week over a 26-week regular season with most teams traveling greater than 40,000 miles over the regular season. The 82-match schedule for each NBA team has been in place since 1967–68 with teams competing four times against opponents in their conference—twice at home and twice away (McLean et al., 2018). Despite the grueling 82-match schedule of a NBA team, very little work has examined the impact of this substantial travel schedule on match performances (Esteves et al., 2020).

Previously, air travel was reported to negatively affect health and recovery for every NBA team (Huyghe et al., 2018). Specifically, these authors indicated that breathing air from a pressurized cabin during air travel may reduce blood oxygen saturation levels leading to a disruption of normal sleep patterns, diet, hydration maintenance, and body rhythms (Huyghe et al., 2018). This reduction in oxygenation, in combination with prolonged sitting during travel, may lead to muscle and joint stiffness that impedes athlete recovery (Leatherwood and Dragoo, 2012). Further, the significant travel schedule may contribute to injuries (McLean et al., 2018) with the number of matches missed by NBA All-Star players (i.e., players who compete in the most matches and travel more than other NBA players) due to injury almost twice as great now as it was in the 1980's (Podlog et al., 2015). The combination of competition and travel was suggested to contribute to a greater risk of bone, joint, and soft tissue injuries as these athletes were more likely to compete with less, and potentially disrupted, rest (Yeh et al., 2012; Podlog et al., 2015; McLean et al., 2018). Indeed, the Golden State Warriors managed their athletes' workloads by having some of them not compete during the fourth quarter or excluding them from some matches during the 2015–2018 seasons, where they exhibited the best win-loss record within the NBA (Zhang et al., 2019a).

While the combination of competition and travel likely impact upon performance within the NBA, the competition schedule or degree of match congestion (e.g., playing matches on consecutive days or back-to-back, playing on 1 day's rest, playing on 2 day's rest, playing on 3 or more day's rest) may also affect performance of NBA teams (Steenland and Deddens, 1997). For example, NBA teams averaged less three-point shots per 100 possessions and 20% less dunks during the fourth quarter compared to the first quarter in a back-to-back series (i.e., matches played on consecutive days) (Steenland and Deddens, 1997). Further, the likelihood of match success increased significantly with 1 day of rest between matches, compared to playing back-to-back matches, with shooting efficacy-related statistics discriminatory of the different match congestion cycles (Esteves et al., 2020). Despite these preliminary studies, it is still unclear how NBA match performances are impacted by back-to-back and various rest-day configuration schedules. Further, identification of the key performance indicators (KPIs) that best differentiate between winning and losing matches during various congestion cycles remains to be confirmed. Understanding how match congestion influences match performances, and their relevant KPIs will support the optimization of team's performance for success.

Using previous work as a starting point, the aim of this study was: (i) to explore the differences in match performances between different match congestion cycles; and (ii) to identify the KPIs discriminating between winning and losing matches under different fixture congestion cycles. It was hypothesized that KPIs for winning and losing would vary with different match congestion cycles and support future strategic frameworks for coaches and teams.

## Materials and Methods

### Sample

This study was a retrospective analysis of publically available data from the NBA official website (http://stats.nba.com/). A total of 1,230 regular season matches were examined during the 2016–2017 NBA season. The first match of the season for each team was not considered due to the lack of a prior match. Our study classified the sample of matches into three types based on the number of rest days (i.e., days with no competitive matches). Back-to-back matches were classified as those without a rest day between matches while 1-day matches included a single rest day between matches. Two-day matches were classified as those that had two or more days of rest between matches. The KPIs were examined according to the situational conditions of offensive and defensive activities (Table 1), and were in accordance with those previously employed (Sampaio et al., 2015b). Furthermore, normalization of all team KPIs was undertaken using the number of ball possessions, as previously described (Leicht et al., 2017). The reliability and validity of the dataset has been previously reported (Zhang et al., 2019a). The current study was conducted according to the ethical guidelines of the authors' affiliated institutions but did not require Ethics Committee approval because a non-interventional design was used, whereby all analyzed data were de-identified and aggregated archival data available in the public domain.

**Table 1:** The variables examined in the current study.

Opponent quality: Strong and weak teams
Match type: Balanced and unbalanced
Match location: Home and away
Match pace: Fast-paced and slow-paced
Paint score: The number of points scored by a team in the keyway, also known as the paint area
Mid-range score: The number of points scored by a team outside of the paint area but inside the three-point line
Three-point score: The number of three-point field-goals that a team scored
Free-throws: The number of free throws that a team scored
Offensive rebounds: The number of rebounds a team collected while they were on offense
Assists: An assist occurs when a player completes a pass to a teammate that directly leads to a field goal score
Turnovers: A turnover occurs when the team on offense loses the ball to the defense
Defensive rebounds: The number of rebounds a team collected while they were on defense
Steals: A steal occurs when a defensive player takes the ball away from a player on offense
Blocks: A block occurs when the defense player tips the ball and prevents an offensive player's shot from scoring
Personal fouls: The total number of fouls that a team committed

In order to control for the situational conditions during different match congestion cycles, opponent quality, match type, match location, and match pace (see below) were considered in our study.

#### Opponent Quality

This was defined using the team's winning match percentage (Gómez et al., 2013). A *k*-means cluster analysis identified two clusters: weak teams (winning = 39.3 ± 7.3%) and strong teams (winning = 60.2 ± 9.2%).

#### Match Type

A *k*-means cluster analysis identified a threshold for final team score/points differences of a match (Zhang et al., 2019b) with balanced (cluster 1, 1–16 points difference) and unbalanced (cluster 2, >16 points difference) matches identified.

#### Match Location

This was defined as the match being played at home or away (Gómez et al., 2010).

#### Match Pace

Balanced matches (previously defined) were divided via a k-means cluster analysis into fast (104.2 ± 2.9 possessions) and slow-paced (95.8 ± 3.2 possessions) matches according to the number of ball possessions (Gómez et al., 2017).

### Statistical Analysis

To address aim 1, the differences in match performance variables between Back-to-back matches, 1-, and 2-day matches were conducted by a one-way ANOVA and pairwise comparisons with Bonferroni correction (Ibanez et al., 2003). Effect size (ES) was calculated to determine the meaningfulness of the differences and magnitudes were expressed as eta-squared (η^2^) with the following threshold values employed: >0.01 (small), >0.06 (moderate), and >0.15 (large) (Girden, 1992).

To address aim 2, binary logistical regression was used to develop a linear probability model with the dependent variable of match outcome set as WIN = 1 and LOSS = 0. All assumptions relating to the use of this statistical approach were met. Odds ratios (OR) and corresponding 95% confidence intervals (95% CI) were obtained in order to provide a standardized measure of the influence of each factor included in the models. Performance of each model was evaluated as the percentage of match outcomes correctly classified. All analyses were undertaken using the R software (R project version 4.0.0) and a level of significance was accepted at *P* ≤ 0.05, unless otherwise indicated.

## Results

Descriptive statistics for KPIs during different match congestion cycles are presented in Table 2. The majority of KPIs were similar between match congestion cycles except for paint score (ES = 0.08) and three-point score (ES = 0.05), which were significantly less for Back-to-back compared to 1- and 2-day matches (Table 2).

**Table 2:** Descriptive statistics for key performance indicators (KPIs) under different match congestion cycles.

**KPIs**	**Back-to-back matches**	**One-day matches**	**Two-day matches**	***F***	***P***	**ES**
Paint score	42.2 ± 9.5	43.6 ± 9.6^a^	44.6 ± 9.9^a^	14.420	*P* = 0.001	0.08
Mid-range score	15.4 ± 7.0	15.2 ± 7.2	15.3 ± 7.0	0.997	*P* = 0.318	0.02
Three-point score	28.7 ± 10.5	28.9 ± 10.4^a^	29.6 ± 10.8^a^	6.440	*P* = 0.011	0.05
Free throws	17.8 ± 6.1	17.9 ± 6.6	17.7 ± 6.3	0.104	*P* = 0.748	0.01
Offensive rebounds	10.1 ± 3.7	10.1 ± 3.8	10.2 ± 3.8	0.044	*P* = 0.834	0.01
Assists	21.9 ± 5.2	22.7 ± 5.2	23.1 ± 5.3	1.270	*P* = 0.260	0.02
Turnovers	13.1 ± 3.8	13.4 ± 3.9	13.5 ± 3.6	2.939	*P* = 0.087	0.04
Defensive rebounds	33.0 ± 5.0	33.5 ± 5.4	33.4 ± 5.3	0.682	*P* = 0.409	0.02
Steals	7.4 ± 2.8	7.8 ± 2.9	7.8 ± 2.8	1.303	*P* = 0.254	0.02
Blocks	4.4 ± 2.3	4.8 ± 2.5	4.7 ± 2.6	2.055	*P* = 0.152	0.03
Personal fouls	20.4 ± 4.3	19.7 ± 4.2	19.8 ± 4.2	6.963	*P* = 0.118	0.01

a *P < 0.05 vs. Back-to-back matches*.

**Figure 1:**
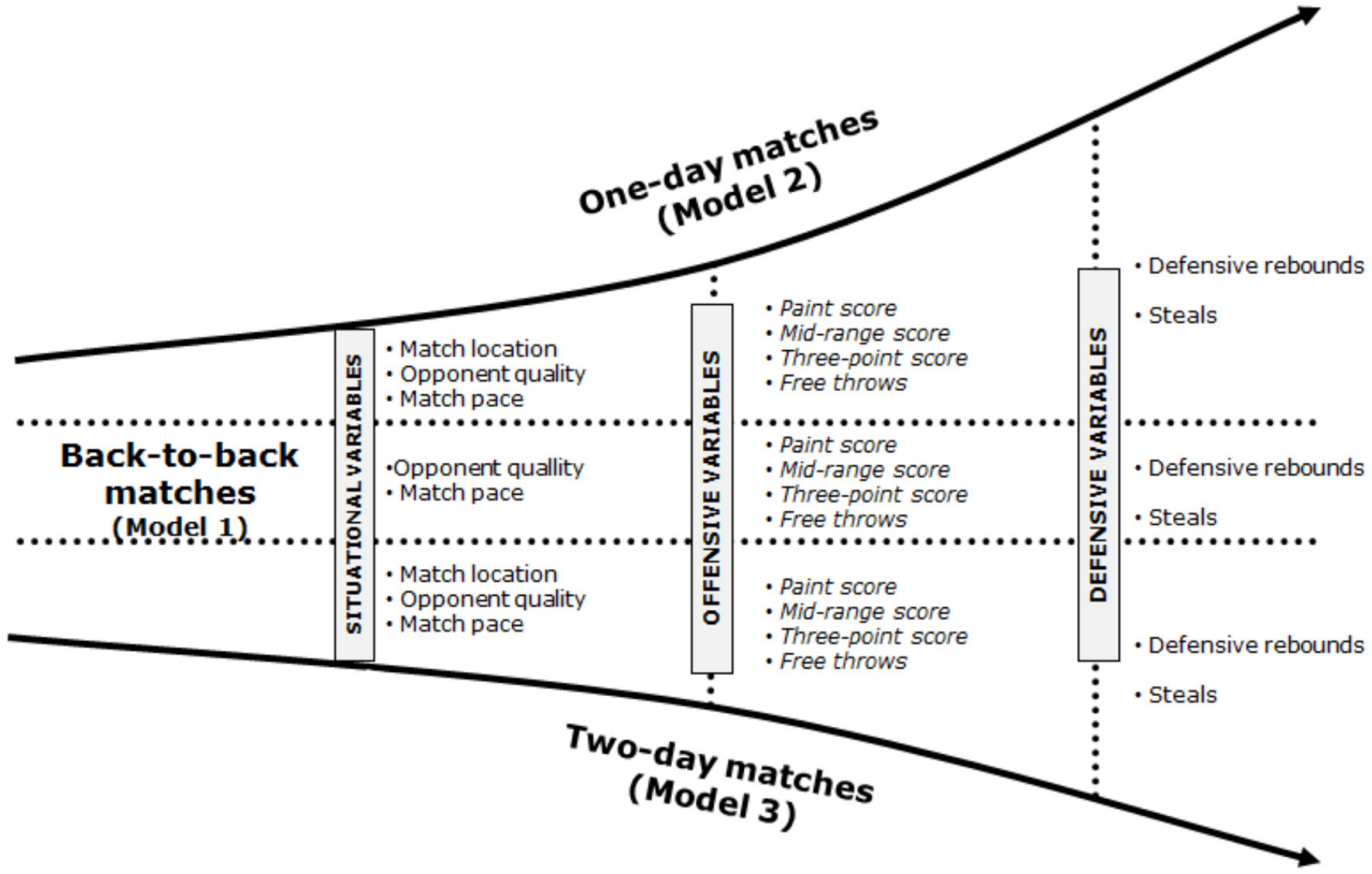
Summary of significant key performances indicators identified via logistical regression modeling for Back-to-back, 1- and 2-day matches.

The binomial logistical regression results are presented in Table 3 with classification accuracies being 79.9, 81.6, and 83.1% for models 1, 2, and 3, respectively. Details of the specific results are presented below with a summary of these shown in Figure 1.

**Table 3:** Results relating to the three logistic regression models developed for Back-to-back, 1-, and 2-day matches.

	**Model 1**						**Model 2**						**Model 3**					
	**Back-to-back matches** ***(WIN/LOSE)***						**One-day matches** ***(WIN/LOSE)***						**Two-day matches** ***(WIN/LOSE)***					
	**β (S.E.)**		**χ^2^**	**OR (95% CI)**		***P***	**β (S.E.)**		**χ^2^**	**OR (95% CI)**		***P***	**β (S.E.)**		**χ^2^**	**OR (95% CI)**		***P***
Constant	−18.935	2.106	80.856			0	−20.357	1.302	244.384			0	−22.168	2.557	75.188			0
Match location (1)	−0.464	0.257	3.260	0.629	(0.380, 1.04)	0.071	−0.370	0.144	6.638	0.691	(0.521, 0.915)	<0.010	−0.666	0.285	5.453	0.514	(0.294, 0.898)	<0.020
Opponent quality (1)	−0.830	0.255	10.616	0.436	(0.265, 0.718)	<0.001	−0.576	0.143	16.174	0.562	(0.425, 0.744)	<0.001	−1.034	0.285	13.131	0.355	(0.203, 0.622)	<0.001
Match type (1)	−0.078	0.363	0.047	0.925	(0.454, 1.883)	0.829	0.048	0.226	0.045	1.049	(0.673, 1.635)	0.832	0.367	0.438	0.702	1.444	(0.611, 3.408)	0.402
Match pace (1)	−1.887	0.346	29.766	0.152	(0.077, 0.298)	<0.001	−1.707	0.189	81.12	0.181	(0.125, 0.263)	<0.001	−1.915	0.383	25.035	0.147	(0.070, 0.312)	<0.001
Paint score	0.116	0.019	35.551	1.123	(1.081, 1.167)	<0.001	0.108	0.011	90.418	1.114	(1.089, 1.139)	<0.001	0.140	0.022	39.097	1.151	(1.101, 1.203)	<0.001
Mid–range score	0.128	0.023	30.512	1.137	(1.086, 1.190)	<0.001	0.132	0.014	90.313	1.141	(1.110, 1.173)	<0.001	0.159	0.028	31.854	1.172	(1.109, 1.238)	<0.001
Three-point score	0.131	0.019	48.626	1.140	(1.099, 1.183)	<0.001	0.135	0.011	146.666	1.144	(1.120, 1.170)	<0.001	0.162	0.022	52.828	1.176	(1.126, 1.229)	<0.001
Free throws	0.163	0.024	45.580	1.177	(1.122, 1.233)	<0.001	0.140	0.014	106.213	1.150	(1.120, 1.181)	<0.001	0.126	0.026	24.227	1.135	(1.079, 1.193)	<0.001
Offensive rebounds	0.017	0.035	0.240	1.017	(0.950, 1.089)	0.624	−0.003	0.020	0.029	0.997	(0.959, 1.036)	0.865	−0.091	0.038	5.701	0.913	(0.848, 0.984)	0.117
Assists	0.022	0.031	0.481	1.022	(0.961, 1.087)	0.488	0.030	0.019	2.623	1.031	(0.994, 1.07)	0.105	−0.022	0.037	0.360	0.978	(0.909, 1.052)	0.549
Turnovers	−0.041	0.037	1.235	0.960	(0.893, 1.032)	0.266	−0.049	0.020	6.158	0.952	(0.916, 0.990)	0.117	−0.021	0.041	0.265	0.979	(0.903, 1.061)	0.607
Defensive rebounds	0.155	0.028	29.878	1.168	(1.105, 1.235)	<0.001	0.230	0.017	178.945	1.259	(1.217, 1.302)	<0.001	0.245	0.034	50.867	1.277	(1.194, 1.366)	<0.001
Steals	0.315	0.054	34.332	1.371	(1.233, 1.523)	<0.001	0.251	0.028	77.625	1.285	(1.215, 1.359)	<0.001	0.315	0.060	27.784	1.370	(1.219, 1.541)	<0.001
Blocks	0.131	0.055	5.563	1.14	(1.022, 1.270)	0.318	0.117	0.03	15.17	1.124	(1.060, 1.192)	0.128	0.035	0.055	0.404	1.036	(0.930, 1.153)	0.525
Personal fouls	−0.089	0.031	8.549	0.915	(0.861, 0.971)	0.361	−0.089	0.018	23.787	0.915	(0.882, 0.948)	0.213	−0.059	0.035	2.806	0.943	(0.883, 1.016)	0.094
Chi–square	4.540[*df*=8]				0.805	17.531[*df*=8]				0.052	15.124 [df = 8]				0.057			
Cases correctly classified			79.90%						81.60%						83.10%			

### Situational Variables

Compared to away matches, teams at home had a 0.514–0.691 times higher likelihood of winning 1- and 2-days matches (*P* < 0.02, Table 3). Match location had no significant impact on winning for Back-to-back matches (Table 3). Compared to weak teams, strong teams had a 0.355–0.562 times higher likelihood of winning Back-to-back, 1-, and 2-day matches (*P* < 0.001, Table 3). Match type (balance or unbalanced) had no significant impact on winning for any match congestion cycle (Table 3). Compared to slow paced, fast-paced matches had a 0.147–0.181 times higher likelihood of winning Back-to-back, 1- and 2-day matches (*P* < 0.001, Table 3).

### Offensive Variables

Shooting ability featured significantly in all three models (Table 3). Specifically, a greater Paint score was associated with a 11, 12, and 15% greater probability of winning 1-day, Back-to-back, and 2-day matches, respectively (*P* < 0.001, Table 3). Likewise, a greater Mid-range score was associated with a ~14, 14, and 17% greater probability of winning Back-to-back, 1-, and 2-day matches, respectively (*P* < 0.001, Table 3). A greater Three-point score was associated with a 14, 14, and ~18% greater probability of winning Back-to-back, 1- and 2-day matches, respectively (*P* < 0.001, Table 3). Finally, greater Free throws was associated with a ~18, 15, and ~14% greater probability of winning Back-to-back, 1- and 2-day matches, respectively (*P* < 0.001, Table 3). No significant associations were identified for Offensive rebounds, Assists, and Turnovers in any regression model (*P* > 0.1, Table 3).

### Defensive Variables

A greater number of Defensive rebounds was associated with a ~17, ~26, and ~28% (*P* < 0.001) greater probability of winning Back-to-back, 1-, and 2-day matches, respectively (*P* > 0.001, Table 3). Similarly, a greater number of Steals was associated with a 37, ~29, and 37% greater probability of winning Back-to-back, 1- and 2-day matches, respectively (*P* > 0.001, Table 3). No significant associations were identified for Blocks or Personal fouls in any regression model (*P* > 0.09, Table 3).

## Discussion

The aims of this study were (i) to explore the differences in match KPIs between different match congestion cycles, and (ii) to identify the KPIs discriminating between winning and losing matches under different fixture congestion cycles. The current findings supported our hypothesis and highlighted Paint score and Three-point score as the KPIs that best discriminated between different match congestion cycles. In addition, shooting ability, Defensive rebounds, and Steals were important KPIs differentiating between winning and losing under different match congestion cycles. Similarly, Opponent quality and Match pace were vital situational variables that impacted upon winning probability. It is worth noting that Match location had an impact on winning for 1- and 2-day matches but not Back-to-back matches. Our current study highlighted that shooting ability and key defensive actions contribute significantly to winning during different match congestion cycles that would assist coaches in their preparations for elite basketball competition.

### Differences in Match Performances Between Match Congestion Cycles

Our study indicated that congested matches have a great impact on match outcome. Specifically, players scored more in the paint and from the three-point line with a 2 day rest interval between matches compared to consecutive matches. These results were aligned with prior studies that reported 1 day of rest between matches had a positive effect on team scoring (i.e., 1.1-point improvement for the home team and 1.6-point improvement for the visitor team) with this effect peaking with 3 days of rest between matches (Steenland and Deddens, 1997; Esteves et al., 2020). Potentially, the immense commitment, strength, and agility effort exhibited during consecutive matches may impact subsequent match performances with insufficient between-match rest possibly leading to subsequent ineffective technical-tactical activities and lower shooting efficiency (Staunton et al., 2018). Furthermore, our study was in line with previous studies who noted that recovery period between matches had a huge impact on long-distance shooting ability of players (i.e., short rest led to reduced ability) (Ibáñez et al., 2008). The reduced Three-point scoring proficiency for Back-to-back matches compared to 1- and 2-day matches in the current study reinforces the impact of short rest on shooting ability. Collectively, our and prior studies have provided coaches with clear evidence for the benefits of rest between matches but also the development of tactical strategies for Back-to-back matches. For example, teams may slow down match pace and employ more half-court movements in back-to back matches to minimize player effort and improve the chances of winning (Zhang et al., 2018).

### Influence of Situational, Offensive, and Defensive Variables on Winning Probability for Match Congestion Cycles

Our study indicated that opponent quality and match pace were significant predictors of match outcome during different match congestion cycles. Previously, opponent quality and match pace was reported to have a clear impact on technical and tactical execution in the NBA (Sampaio et al., 2015b). For example, successful and better teams in the NBA displayed strong team cooperation and positive tactical intention or offensive involvement, which led to a higher winning probability than weaker teams (Zhang et al., 2017, 2018). In addition, compared to slow paced, fast-paced matches had a higher impact on winning Back-to-back, 1-, and 2-day matches. Coaches should pay more attention to transition/counterattacking play that generates more efficacy and are more unpredictable than organized attacks due to their spontaneity (Ibanez et al., 2019). A defense must be focused on what generates losses or defensive rebounds, as these are the actions that lead to transition/counterattacking play (Ibáñez et al., 2008). Interestingly, our study, like another (Zhang et al., 2017), identified that home matches resulted in a greater winning probability than away matches, most likely due to an important psychological effect on players at home (Pollard et al., 2007). However, this effect was only apparent for 1- and 2-day matches with no home advantage effect for Back-to-back matches. A possible explanation could be that physical and mental fatigue during Back-to-back matches lead to variations in technical actions and tactical coordination errors (Ribeiro et al., 2016).

In terms of offensive KPIs, the current study indicated that greater shooting proficiency, and accumulation of Defensive rebounds and Steals enhanced winning probability during all examined match congestion cycles. Previously, Gómez et al. (2008) and Ibanez et al. (2019) stated that the nature of basketball was to try and score more points than the opposing team. Therefore, it was not surprising that shooting proficiency was a crucial KPI for match success in the current study. However, this study demonstrated that this contribution was apparent regardless of match congestion cycle and therefore, an important focus for athletes and coaches to manage for match success. Subsequently, coaches should ensure that teams possess players with high shooting potential and shooting efficiency throughout the season. For the defensive KPIs, our study was consistent with previous studies (Zhang et al., 2019b) that reported the importance and effectiveness of defensive strategies (i.e., force missed shots that lead to defensive rebounds, and steals) that applied constant pressure on opponents to support their winning. Indeed, several studies have emphasized that an optimal defensive system: (i) can control the pace of play by forcing the opponent's attack to play outside of their usual rhythm of play; (ii) can prevent the best scoring options of the opposing team during competition by applying defensive pressure or stopping individual's attack and passes; and (iii) can extend defenses to a full court range to delay the ball transition and impair the opponents' offensive concentration (Gómez et al., 2010; García et al., 2014; Sampaio et al., 2015a). Importantly, this system of aggressive defense relies upon superior player fitness with high-level defensive performances requiring greater energy demands and greater reaction ability, speed, and repeated high-intensity sprint ability of players (Gómez et al., 2006).

Although the current study provided novel findings, some limitations should be acknowledged for consideration in future research. First, the overall sample size was relatively modest over one season despite being from an elite competition. Future research could expand the sample with a longitudinal design to explore the influence of KPIs on different match congestion cycles across several NBA seasons. Second, the current study considered the contextual effects in isolation on match outcome during different match congestion cycles. Future studies are recommended to examine the possible interaction effects of situational variables on match outcome. Finally, our analyses focused on a senior male competition only with future studies encouraged to examine other competitions (e.g., female, under-18, etc.). Such work will likely extend upon the current results and applicability of modeling technical performances under different match congestion cycles to support coaches for success.

## Conclusion

In summary, Paint score and Three-point score were the KPIs that best discriminated between different match congestion cycles. Importantly, the current study identified shooting efficiency and aggressive defensive strategies (e.g., “Defensive rebounds,” “Steals”) as essential for match success during different match congestion cycles. Opponent quality and match pace were important situational variables that affected winning probability while match location had an impact on match success only for 1- and 2-day matches. Management of these situational and KPIs can provide coaches and teams with a greater probability of winning matches. The development of key offensive and defensive strategies and/or the selection of athletes highly proficient in shooting and aggressive defensive behaviors would likely lead to match success in the NBA.

## Data Availability Statement

The raw data supporting the conclusions of this article will be made available by the authors, without undue reservation.

## Author Contributions

SZ: conceptualization, formal analysis, and visualization. JY and SZ: methodology. CZ: software, investigation, resources, data curation, and project administration. CZ and SZ: validation and supervision. JY: writing—original draft preparation and funding acquisition. M-ÁG and AL: writing—review and editing. All authors have read and agreed to the published version of the manuscript.

## Conflict of Interest

The authors declare that the research was conducted in the absence of any commercial or financial relationships that could be construed as a potential conflict of interest.
